# Postdromal symptoms in migraine: a REFORM study

**DOI:** 10.1186/s10194-024-01716-3

**Published:** 2024-02-21

**Authors:** Janu Thuraiaiyah, Håkan Ashina, Rune Häckert Christensen, Haidar M. Al-Khazali, Messoud Ashina

**Affiliations:** 1 Danish Headache Center, Department of Neurology, Rigshospitalet – Glostrup, Faculty of Health and Medical Sciences, University of Copenhagen, 2600 Glostrup, Denmark; 2 Department of Clinical Medicine, Faculty of Health and Medical Sciences, University of Copenhagen, Copenhagen, Denmark; 3 Department of Brain and Spinal Cord Injury, Copenhagen University Hospital–Rigshospitalet, Copenhagen, Denmark; 4 Harvard Medical School, Boston, MA USA; 5 Department of Anesthesia, Critical Care and Pain Medicine, Beth Israel Deaconess Medical Center, Harvard Medical School, Boston, MA USA; 6 Danish Knowledge Center On Headache Disorders, Glostrup, Denmark

**Keywords:** Migraine hangover, Tiredness, Premonitory, Disease burden

## Abstract

**Background:**

Migraine is a multiphasic neurovascular disorder, where headache can be succeeded by postdromal symptoms. However, there are limited research on postdromal symptoms. This study aimed to investigate the proportion of individuals with migraine from a tertiary care unit reporting postdromal symptoms in adherence with the ICHD-3 definition. We also aimed to examine how the means of enquiry might influence the estimated proportions. Additionally, we explored whether any clinical features might affect the likelihood of reporting postdromal symptoms. Finally, we assessed to what extend the postdromal symptoms might impact the disease burden.

**Methods:**

In a cross-sectional study, we enrolled adult participants diagnosed with migraine who were asked to report their postdromal symptoms (i.e., unprompted reporting). Subsequently, a 16-item list was used to further ascertain the occurrence of postdromal symptoms (i.e., prompted reporting). Clinical characteristics were obtained through a semi-structured interview. Moreover, electronic questionnaires were used to assess the disease burden, i.e., the Six-Item Headache Impact Test (HIT-6), Migraine Disability Assessment (MIDAS), and the World Health Organization Disability Assessment 2.0 (WHODAS 2.0).

**Results:**

Among 631 participants with migraine, a higher proportion experienced at least one postdromal symptom when prompted (*n* = 509 [80.7%]) compared with unprompted reporting (*n* = 421 [66.7%], *P* < 0.001). Furthermore, the total number of postdromal symptoms experienced was greater with prompted than unprompted reporting (medians 3 [IQR 1 – 6] versus 1 [IQR 0 – 2]; *P* < 0.001). Furthermore, the likelihood of reporting postdromal symptoms increased with the presence of premonitory symptoms and decreased with higher number of monthly migraine days. Weak correlations were identified between the number of postdromal symptoms reported and both HIT-6 (*ρ* = 0.14; *P* < 0.001) and WHODAS scores (*ρ* = 0.15; *P* < 0.001), whilst no correlation was observed with MIDAS score (*ρ* = 0.08; *P* = 0.054).

**Conclusions:**

Postdromal symptoms are prevalent in individuals with migraine from a tertiary care unit. However, reported estimates warrant cautious interpretation as they depend on the means of enquiry, presence of premonitory symptoms, and frequency of monthly migraine days. Moreover, a weak correlation was identified between the number of postdromal symptoms and both HIT-6 and WHODAS scores, indicating only a marginal influence on the disease burden.

**Graphical Abstract:**

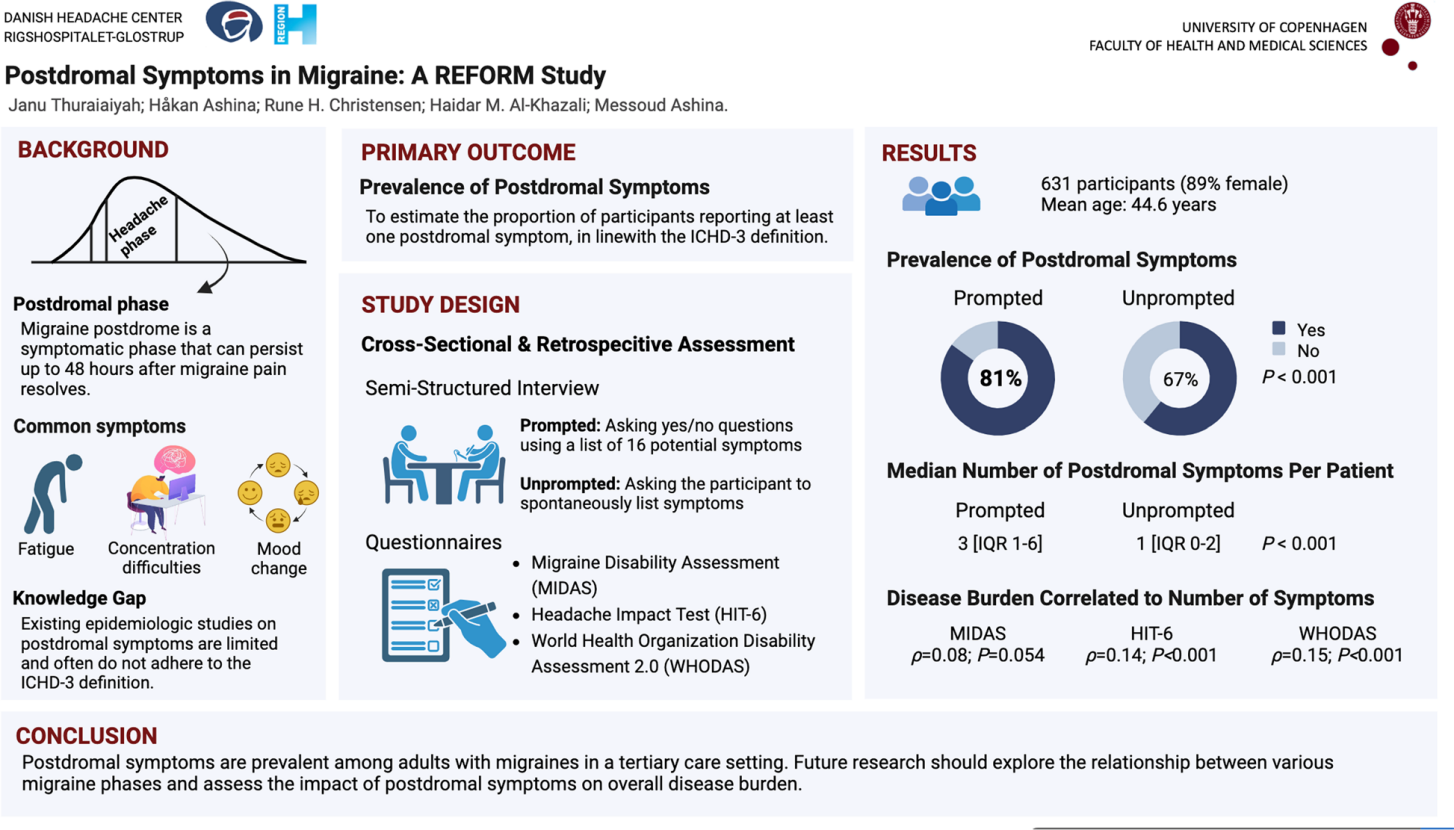

**Supplementary Information:**

The online version contains supplementary material available at 10.1186/s10194-024-01716-3.

## Introduction

Migraine is a disabling neurologic disorder, afflicting more than one billion people worldwide [21]. Central to our current understanding is the description of migraine in distinct phases: interictal, prodrome, aura, headache, and postdrome [6]. Most research has been directed towards dissecting the prodromal (i.e., premonitory), aura, and headache phases, whereas the postdromal phase has historically received less attention.

The postdromal phase, often referred to as the “migraine hangover”, represents the aftermath of a migraine attack [1]. According to the 3rd edition of the International Classification of Headache Disorders (ICHD-3), these symptoms must occur within 48 h after the resolution of pain in migraine attacks [24]. Common postdromal symptoms include tiredness, concentrations difficulties, and mood changes [4]. However, available epidemiologic literature regarding these symptoms is sparse and has not applied the ICHD-3 definition [4]. Other important considerations also remain unanswered. For instance, it needs to be determined whether the means of enquiry influence the reported count of postdromal symptoms. In addition, potential interrelationships between various migraine phases continue to be enigmatic [10]. Lastly, a clear grasp of postdromal symptoms and their impact on the disease burden might shed light on a possibly overlooked but essential aspect of migraine.

To address gaps in our current knowledge, we performed a cross-sectional study involving a large sample of adult participants with migraine. Our results stand as the first to examine the estimated proportion of postdromal symptoms using the ICHD-3 definition. Furthermore, we investigated whether the likelihood of reporting postdromal symptoms is associated to specific clinical features, including other phases of migraine. Finally, we aimed to explore the impact of postdromal symptoms on the disease burden attributable to migraine.

## Methods

The present investigation is part of the larger parental Registry for Migraine (REFORM) study and was carried out at a single tertiary care unit [11]. The study protocol received approval from the relevant ethics committee and data protection agency. Furthermore, the study was conducted in adherence with the principles outlined in Helsinki Declaration, 7th revision [25]. All participants provided written informed consent before any study-related tasks or procedures were performed. A detailed description of the methods used in the parental study can be found elsewhere [11].

### Design

The results presented herein are based on cross-sectional, retrospective observational data. The primary recruitment source was adult individuals diagnosed with migraine from the outpatient clinic of a single tertiary care unit. The enrolment period spanned from September 2020 to June 2022. Potential participants underwent an initial screening via phone. Following this, eligible participants were invited for an on-site visit. Each participant underwent a neurologic examination and a semi-structured interview. Furthermore, they were asked to complete various electronic patient-reported outcome measures (PROMs).

### Participants

Eligible participants were individuals aged 18 years and above, diagnosed with either migraine with or without aura in accordance with the ICHD-3 criteria [24]. Furthermore, participants were required to have experienced, on average, a minimum of 4 monthly migraine days in the three months before enrolment. The key exclusion criteria were onset of migraine after 50 years of age, history of cluster headache or hemiplegic migraine, inability to distinguish migraine from other types of headaches, and being pregnant or breastfeeding. For the present investigation, we also excluded individuals who experienced continuous and unremitting daily headache. For a complete list of inclusion and exclusion criteria, please refer to Supplemental Table 1.

**Table 1:** Demographics and baseline characteristics of the study population

**Demographics and Clinical Characteristics**			
	**Reporting ≥ 1 postdromal symptoms**		
	Yes *n* = 511	No *n* = 120	Overall *n* = 631
Gender, n (%)			
Female	464 (90.8%)	98 (81.7%)	562 (89.1%)
Male	47 (9.2%)	22 (18.3%)	69 (10.9%)
Age, mean (SD)	44.9 (11.8)	43.4 (12.9)	44.6 (12.0)
BMI, mean (SD)	25.0 (4.9)	25.0 (5.2)	25.0 (5.0)
Race, n (%)			
White	469 (91.8%)	115 (95.8%)	584 (92.6%)
Middle Eastern or North African	31 (6.1%)	5 (4.2%)	36 (5.7%)
Black	6 (1.2%)	0 (0%)	6 (1.0%)
Asian or Pacific Islander	4 (0.8%)	0 (0%)	4 (0.6%)
Hispanic or Latin American	1 (0.2%)	0 (0%)	1 (0.2%)
Native American	0 (0%)	0 (0%)	0 (0%)
Headache Diagnoses, n (%)^a^			
Migraine without Aura	502 (98.2%)	118 (98.3%)	620 (98.3%)
Migraine with Aura	166 (32.5%)	32 (26.7)	198 (31.4%)
Chronic Migraine	300 (58.7%)	80 (66.7%)	380 (60.2%)
Medication Overuse Headache	179 (35.0%)	43 (35.8%)	222 (35.2%)
Headache Frequency, mean (SD)			
Monthly Migraine Days	13.7 (6.1)	18.6 (9.4)	14.6 (7.1)
Total Headache Days Per Month	17.2 (6.5)	20.8 (8.4)	17.9 (7.1)
Migraine Intensity, n (%)			
Mild	1 (0.2%)	2 (1.7%)	3 (0.5%)
Moderate	148 (29.0%)	37 (30.8%)	185 (29.3%)
Severe	362 (70.8%)	81 (67.5%)	443 (70.2%)
Clinical Features, n (%)			
Pulsating	392 (76.7%)	87 (72.5%)	479 (75.9%)
Pressing	226 (44.2%)	65 (54.2%)	291 (46.1%)
Aggravated by physical activity	465 (91.0%)	106 (88.3%)	571 (90.5%)
Presence of Nausea	481 (94.1%)	107 (89.2%)	588 (93.2%)
Presence of Photophobia	492 (96.3%)	112 (93.3%)	604 (95.7%)
Presence of Phonophobia	481 (94.1%)	105 (87.5%)	586 (92.9%)
Presence of Autonomic Symptoms	291 (56.9%)	51 (42.5%)	342 (54.2%)
Current Use of Treatment, n (%)			
Current Use of Acute Medication	501 (98.0%)	115 (95.8%)	616 (97.6%)
Current Use of Triptans	469 (91.8%)	101 (84.2%)	570 (90.3%)
Current Use of Preventive Medication	291 (56.9%)	59 (49.2%)	350 (55.5%)
Reports Premonitory Symptoms, n (%)	413 (80.8%)	34 (28.3%)	447 (70.8%)
Reports Trigger Factors, n (%)	493 (96.5%)	113 (94.2%)	606 (96.0%)

*BMI* Body Mass Index, *SD* Standard Deviations

^a^Headache diagnoses were not mutually exclusive. Participants experiencing migraine with aura (MA) and without aura (MO) were categorized under both MA and MO. Those meeting the criteria for chronic migraine (CM), based on headache frequency, and medication-overuse headache (MOH), due to acute medication use, were concurrently assigned the diagnoses of MA and/or MO, CM, and MOH. This approach was adopted to comprehensively represent the diverse migraine presentations within our study population

### Procedures

The semi-structured interview was conducted to obtain demographics, headache characteristics, and full medical history. Within the course of the interview, participants were educated on the distinct phases of a migraine attack. This was followed by a series of questions about both premonitory and postdromal symptoms. To ascertain the latter, participants were asked to list all symptoms manifesting within 48 h after the resolution of pain in a migraine attack (i.e., unprompted reporting). If clarity was required, the question was rephrased or explained using a drawing of the migraine phases, omitting mention of specific symptoms. Following this, participants were enquired regarding the occurrence of 16 pre-defined symptoms using close-ended questions (prompted reporting). Participants were also offered the option to introduce additional symptoms. Data regarding premonitory symptoms and triggers factors were also obtained in a similar manner. In addition, participants were asked about comorbid conditions – these were verified using electronic medical records when available.

PROMs were administered and stored using the Research Electronic Data Capture (REDCap) software. The administered questionnaires comprised the Migraine Disability Assessment (MIDAS), Six-Item Headache Impact Test (HIT-6), World Health Organization Disability Assessment 2.0 (WHODAS 2.0), alongside other PROMs reported elsewhere [11].

### Definitions

Postdromal symptoms were defined in accordance with the ICHD-3 definition [24]. The latter characterizes postdromal symptoms, lasting up to 48-h, and follows the resolution of pain in a migraine attack with or without aura. Moreover, premonitory symptoms were defined as symptoms occurring 2 to 48 h prior to the onset of pain in a migraine attack. This definition aligns with the description in both ICHD-2 and ICHD-3β [26, 27].

### Migraine disability assessment

The MIDAS questionnaire is a self-report instrument, evaluating headache-related disability in individuals with migraine [20]. MIDAS accounts for the number of days on which work/school, household chores, or non-work activities were prevented or impaired due to headache. The appraisal period is the past 90 days. The calculated score can be stratified into little or no disability (0–5), mild disability (6–10), moderate disability (11–20) and severe disability (≥ 21). MIDAS’ upper limit is a score of 450.

### Headache impact test

HIT-6 is a self-report instrument with six items, assessing headache-related burden [23]. The appraisal period is the past four weeks for the final three questions, while there is no explicit appraisal period for the remaining questions. Each item is scored from 6 to 13, with a total score ranging from 36 to 78 points. The total score can then be categorized into little or no impact (≤ 49), some impact (50–59), substantial impact (56–59) and severe impact (60–78).

### World Health Organization disability assessment schedule 2.0

The WHODAS 2.0 questionnaire is a 12-item instrument to assess disability due to health conditions [5]. The appraisal period is the past 30 days, and each question is rated on a 5-point scale, ranging from “not at all” to “extremely difficult”. The total score ranges from 12 to 60 points.

### Outcomes

The primary objective was to estimate the proportion of participants listing postdromal symptoms (i.e., ≥ 1 symptom) using prompted reporting and in adherence with the ICHD-3 definition [24]. The secondary objectives were exploring differences between prompted and unprompted reporting in terms of the proportion of participants with ≥ 1 postdromal symptom. A similar comparison was made for the total number of postdromal symptoms per participant using the two means of enquiry. Another objective was to examine whether the likelihood of reporting postdromal symptoms was affected by certain clinical factors. This included age, gender, migraine with aura, chronic migraine, medication-overuse headache (MOH), monthly migraine days, migraine duration, migraine severity, current use of triptans, current use of preventive migraine medication, premonitory symptoms, and trigger factors. Moreover, we explored whether MIDAS, HIT-6 and WHODAS 2.0 scores/grades differed between participants with and without postdromal symptoms. Finally, we assessed if correlations existed between the number of postdromal symptoms with the total score of MIDAS, HIT-6, and WHODAS 2.0 individually.

### Statistical analysis

Participant demographics and clinical characteristics were summarized using descriptive statistics. Categorical data are presented as frequency counts (n) and percentages (%). The distribution of continuous data was assessed for normality using the Kolmogorov–Smirnov test. Means and standard deviations (SD) were then used for data following a normal distribution, while medians and interquartile ranges (IQR) were used for skewed data.

McNemar’s test was used for analyses of paired nominal data, while the Mann–Whitney U test was performed non-parametric continuous data. For categorical data involving three or more groups, Fisher’s exact test was used. Correlation analyses were performed using Spearman’s rank correlation and presented with the correlation coefficient, *ρ*, and *P* values. To assess factors that influence the likelihood of reporting postdromal symptoms, we applied a 2-step approach. First, each pre-defined factor was assessed using logistic regression. Those that were significant, exhibiting *P* values ≤ 0.05, were then tested using binominal logistic regression and reported as odds ratios (ORs) with 95% confidence intervals (95% CI) and corresponding *P* values. The Bonferroni correction was applied to the binominal logistic regression. The threshold for statistical significance was set at *P*-values ≤ 0.05. All analyses were performed in *R* (version 4.2.0).

## Results

### Participants

A total of 633 adult individuals with migraine participated in the study. Of these, two did not provide information on postdromal symptoms, which rendered data from 631 participants suitable for analyses. The mean participant age was 44.6 (SD, 12.0) years, with most being female (*n* = 562 [89%]).

The most common diagnosis was migraine without aura (*n* = 620 [98%]), but 198 of these participants also reported migraine with aura, while 11 participants reported experiencing only migraine with aura. Furthermore, 380 (60%) participants had chronic migraine and 222 (35%) participants met the ICHD-3 criteria for MOH. Current use of triptans was reported by 570 (90%) participants, and 350 (55%) were on preventive migraine medication. Regardless of the assessment method, whether prompted or unprompted, 447 (71%) participants reported experiencing premonitory symptoms, whereas 511 (81%) documented postdromal symptoms. Table 1 provides a detailed overview of participant demographics and clinical characteristics.

### Postdromal symptoms

Of 631 participants, 509 (80.7%) noted experiencing ≥ 1 postdromal symptom when presented with a pre-defined list of symptoms (prompted reporting). In contrast, 421 (66.7%) experienced ≥ 1 postdromal symptom when the enquiry was unprompted (*P* < 0.001; Table 2). The mean number of postdromal symptoms also differed between prompted and unprompted reporting (medians 3 [IQR 1 – 6] versus 1 [IQR 0 – 2]; *P* < 0.001; Table 2). Furthermore, all individual postdromal symptoms were more often reported with prompting than without (*P* < 0.001 for all tests).

**Table 2:** Relative frequency of postdromal symptoms reported unprompted and prompted

Postdromal symptom (PS)	Unprompted (*n* = 631)	Prompted (*n* = 631)	*P-value*
Reported PS (≥ One Symptom)	421 (66.7%)	509 (80.7%)	< *0.001*
Number of Symptoms Reported, Median [IQR]	1 [0–2]	3 [1–6]	< *0.001*
Reported one PS, n (%)	157 (24.9%)	86 (13.6%)	
Reported two PS, n (%)	128 (20.3%)	78 (12.4%)	
Reported three PS, n (%)	75 (11.9%)	64 (10.1%)	
Reported four PS, n (%)	35 (5.5%)	69 (10.9%)	
Reported five or more PS, n (%)	26 (4.1%)	212 (33.6%)	
Specific symptoms, n (%)			
Difficulty Concentrating	58 (9.2%)	213 (33.8%)	< *0.001*
Difficulty Writing and Reading	2 (0.3%)	61 (9.7%)	< *0.001*
Dizziness	17 (2.7%)	57 (9.0%)	< *0.001*
Facial Paleness	1 (0.2%)	81 (12.8%)	< *0.001*
Hunger	58 (9.2%)	155 (24.6%)	< *0.001*
Irritability	21 (3.3%)	123 (19.5%)	< *0.001*
Mood Changes	67 (10.6%)	159 (25.2%)	< *0.001*
Nausea or Vomiting	35 (5.5%)	84 (13.3%)	< *0.001*
Neck Pain	29 (4.6%)	142 (22.5%)	< *0.001*
Phonophobia	22 (3.5%)	123 (19.5%)	< *0.001*
Photophobia	28 (4.4%)	137 (21.7%)	< *0.001*
Frequency of Urination	6 (1.0%)	57 (9.0%)	< *0.001*
Tendency Sweating	2 (0.3%)	28 (4.4%)	< *0.001*
Thirst	35 (5.5%)	192 (30.4%)	< *0.001*
Tiredness	317 (50.2%)	426 (67.5%)	< *0.001*
Urge to Yawn	3 (0.5%)	76 (12.0%)	< *0.001*
Other	146 (23.1%)	38 (6.0%)	< *0.001*

*IQR* Interquartile ranges, *PS* Postdromal symptom(s)

When using prompted reporting, the three most common postdromal symptoms were tiredness (*n* = 426 [67.5%]), concentrations difficulties (*n* = 213 [33.8%]), and thirst (*n* = 192 [30.4%]). Unprompted reporting, in turn, revealed tiredness (*n* = 317 [50.2%]) to be the most prevalent postdromal symptom followed by mood changes (*n* = 67 [10.6%]). Beyond the pre-defined list of 16 individual symptoms, an additional 38 distinct symptoms were reported unprompted.

### Associated factors

Logistic regression analyses revealed several clinical factors associated with the likelihood of experiencing postdromal symptoms. Foremost among these was reporting premonitory symptoms, with an odds ratio of 10.66 (95% CI: 6.83–16.97; *P* < 0.001). Also being female carried an increased likelihood of having postdromal symptoms (OR: 2.22, 95% CI: 1.26–3.81; *P* = 0.005), as did reporting current use of triptans (OR: 2.10, 95% CI: 1.15–3.72; *P* = 0.013). Conversely, a higher number of monthly migraine days inversely affected the likelihoods of reporting postdromal symptoms (OR: 0.91, 95% CI: 0.89–0.94; *P* < 0.001). The other factors evaluated did not reach a level of statistical significance, as detailed in Table 3.

**Table 3:** Presence of postdromal symptoms and risk factors

	**Simple Logistic Regression (Univariate)**		**Binomial Logistic Regression (Multivariate)**	
	**Odds Ratio (95% CI)**	*P-value*	**Odds Ratio (95% CI)**	*P-value*
Age	1.01 (0.99–1.02)	0.21		
Female	2.22(1.26–3.81)	0.005	2.09 (1.08–3.96)	0.03
Migraine With Aura	1.32 (0.86–2.09)	0.22		
Chronic Migraine	0.80 (0.52–1.22)	0.31		
Medication-Overuse Headache	0.97 (0.64–1.47)	0.87		
Monthly Migraine Days	0.91 (0.89–0.94)	< 0.001	0.94 (0.91–0.97)	< 0.001^*^
Migraine Duration	1.00 (0.998–1.01)	0.15		
Migraine Intensity (NRS score)	1.06 (0.93–1.22)	0.37		
Current Use of Triptans as Acute Treatment	2.10 (1.15–3.72)	0.013	1.14 (0.55–2.28)	0.72
Current Use of Preventive Medication	1.37 (0.92–2.04)	0.12		
Presence of Premonitory Symptoms	10.66 (6.83–16.97)	< 0.001	8.77 (5.53–14.14)	< 0.001^*^
Presence of Trigger Factors	1.70 (0.65–4.00)	0.25		

Each clinical factor was assessed using simple logistic regression, and those that exhibiting *P* values ≤ 0.05, were then tested using binominal logistic regression. The Bonferroni correction was applied to the binominal logistic regression. *NRS* Numerical Rating Scale

^*^Significant following Bonferroni correction

The identified significant factors were then tested using binomial logistic regression. Among them, three factors remained significantly associated with reporting postdromal symptoms. These were experiencing premonitory symptoms (OR: 8.77, 95% CI: 5.53–14.14; *P* < 0.001), being female (OR: 2.09, 95% CI: 1.08–3.96; *P* = 0.03), and the number of monthly migraine days (OR: 0.94, 95% CI: 0.91–0.97; *P* < 0.001). However, current use of triptans did not remain significant (OR: 1.14, 95% CI: 0.55–2.28; *P* = 0.72). After Bonferroni correction, significant associations were only present for reporting premonitory symptoms and the number of monthly migraine days.

### Burden measures

For the MIDAS questionnaire, 466 participants (91.2%) with postdromal symptoms and 112 participants (93.3%) without such symptoms responded (Table 4). The median MIDAS scores did not differ between these groups: 53.0 [IQR 29.0–96.0] points for participants with symptoms and 69.5 [IQR 26.8–112.0] points for those without (*P* = 0.09). The lack of statistical significance remained even after adjusting for the number of monthly migraine days (*P* < 0.001) and the presence of comorbid chronic pain conditions (*P* = 0.01). The MIDAS grades also did not differ based on the presence of postdromal symptoms (*P* = 0.62). Moreover, the MIDAS scores did not correlate the number of postdromal symptoms reported, both when assessed prompted (*ρ* = 0.08; *P* = 0.054) or unprompted (*ρ* = 0.005; *P* = 0.90).

**Table 4:** Postdromal symptoms and migraine burden

	**Reporting postdromal symptoms**		***P-value***
	Yes	No	
**MIDAS**			
Number of Subjects with Replies	466	112	
Median score [IQR]	53.0 [29.0–96.0]	69.5 [26.8–112.0]	0.09
Grading, n (%)			0.62
No or Little Disability	9 (1.9%)	4 (3.6%)	
Mild Disability	15 (3.2%)	4 (3.6%)	
Moderate Disability	54 (11.6%)	8 (7.1%)	
Severe Disability	388 (83.3%)	96 (85.7%)	
**HIT-6**			
Number of Subjects with Replies	470	112	
Median score [IQR]	64 [62–66]	64 [61.8–67]	0.68
Grading, n (%)			0.71
No or Little Disability	1 (0.2%)	1 (0.9%)	
Mild Disability	17 (3.6%)	5 (4.5%)	
Moderate Disability	37 (7.9%)	10 (8.9%)	
Severe Disability	415 (88.3%)	96 (85.7%)	
**WHODAS**			
Number of Subjects with Replies	416	112	
Median Score [IQR]	21 [16–27]	22 [16–29]	0.47

*HIT-6* Six-Item Headache Impact Test, *IQR* Interquartile ranges, *MIDAS* Migraine Disability Assessment, *WHODAS* World Health Organization Disability Assessment

For the HIT-6 questionnaire, 470 (92.0%) participants with postdromal symptoms and 112 (93.3%) without such symptoms provided responses (Table 4). The median HIT-6 scores were comparable between these groups; 64 (IQR 62.0–66.0) points for those with symptoms and 64 (IQR 61.8–67) points for those without (*P* = 0.68). This non-significance persisted, even after adjusting for the number of monthly headache days (*P* < 0.001) and comorbid chronic pain conditions (*P* = 0.51). The HIT-6 grades also did not differ between the two groups (*P* = 0.71). Moreover, a weak correlation was identified between the HIT-6 scores and the number of postdromal symptoms reported when prompted (*ρ* = 0.14; *P* < 0.001), but not unprompted (*ρ* = 0.06; *P* = 0.17).

For the WHODAS 2.0 questionnaire, 416 (81.4%) participants with postdromal symptoms and 112 (93.3%) without such symptoms responded (Table 4). The median WHODAS scores did not differ between these two groups (22 [16–29] vs. 21 [16–27]; *P* = 0.47). This remained insignificant after adjusting for the number of monthly headache days (*P* < 0.001), comorbid chronic pain conditions (*P* < 0.001), current major depressive disorder (*P* < 0.001) and current anxiety disorder (*P* = 0.17). Moreover, the WHODAS score correlated weakly with the number of postdromal symptoms reported prompted (*ρ* = 0.15; *P* < 0.001). No such correlation was observed with the corresponding unprompted responses (*ρ* = 0.02; *P* = 0.56).

## Discussion

The present study offers novel insights into the migraine postdrome and stands as the first investigation to use the ICHD-3 definition of postdromal symptoms. Our findings reveal a high prevalence of these symptoms among adults with migraine from a tertiary care population. Approximately 80% of our participants reported experiencing postdromal symptoms, with the most common ones being tiredness and concentration difficulties. These observations accord well with estimates from a recent meta-analysis [4] and support our clinical impression that migraine is not limited to the headache phase. It seems evident that various symptoms present themselves both before (i.e., premonitory) and after (i.e., postdromal) the actual migraine pain.

### Assessing postdromal symptoms

Another important finding is the discrepant reporting of postdromal symptoms when assessed prompted versus unprompted. Our results reveal that the relative frequency pertaining to both the total number of symptoms and each individual symptom is greater when assessed prompted than unprompted. One explanation might be that most do experience postdromal symptoms but fail to associate these symptoms with their migraine. However, prompted reporting carries the risk of acquiescence bias [2]. It is also worth noting that the means of enquiry similarly impacts the reporting of premonitory symptoms in individuals with migraine from the same cohort [28]. Taken together, a standardized assessment method is warranted to aid recognition of postdromal symptoms while minimizing the risk of acquiescence bias and false attribution.

In contrast to previous studies that used a 24-h limit [16, 18], our investigation evaluated postdromal symptoms presenting up to 48 h after the resolution of headache. It is therefore interesting that despite our extended duration, the proportion of individuals reporting postdromal symptoms accords well with previous studies [4]. This indicates that the onset of postdromal symptoms most often occur within 24 h after the resolution of headache. However, the duration of these symptoms might still extend beyond 24 h. One previous investigation reported that about one-third of participants with migraine experienced postdromal symptoms lasting more than 24 h [15]. Another retrospective, cross-sectional study found that postdromal symptoms persisted on average for 25.2 h [13]. However, some important questions remain unanswered and warrant further investigation. The onset and duration of individual postdromal symptoms needs to be firmly established. In this context, future enquires should also ascertain whether certain postdromal symptoms manifest in antecedent phases of migraine and then persist in subsequent phases. Such continuity, if confirmed, invites a more nuanced perspective on the description of migraine phases.

### Associated factors

Our findings revealed an inverse relationship between the number of monthly migraine days and the reporting of postdromal symptoms. In addition, the presence of premonitory symptoms increased the likelihood of reporting postdromal symptoms. These observations seem conceivable, as high-frequency migraine renders it difficult to distinguish between different phases of migraine. The ICHD-3 indeed also states that for high-frequency migraine, it can be impossible in some cases to distinguish one attack from another. Thus, future studies might consider exclusion of individuals with high-frequency migraine to accurately assess the occurrence of premonitory and postdromal symptoms. However, a growing body of evidence indicate that non-headache symptoms can persist throughout the premonitory, headache, and postdromal phases of migraine [3, 9, 12, 16]. This continuity not only reaffirms the clinical similarities but also alludes to a shared pathophysiologic substrate across different phases of migraine.

### Burden measures

Multiple studies have found that the migraine postdrome has negative effects on patients’ daily functioning and overall quality of life [3, 9, 15, 22]. Our study revealed a weak positive correlation between the number of postdromal symptoms reported prompted and both HIT-6 and WHODAS scores. It is also worth noting that no such correlation was observed with the MIDAS scores, which is congruent with a previous investigation [18]. The discrepant findings between MIDAS and HIT-6 might be attributed to their different objectives. MIDAS quantifies days missed or with reduced productivity due to migraine, while HIT-6 considers the broader impact of headache on quality of life, encompassing factors such as pain, social and cognitive functioning, and physiological distress [19].

From a patient perspective, the adverse effects of migraine also extend into the postdromal phase, negatively impacting work, social interactions, and family life [3, 15, 17]. Indeed, a large online survey revealed that about half of individuals with migraine felt very or extremely limited in completing daily tasks during the postdromal phase [8]. Another study found that those with postdromal symptoms tend to report increased work or school absenteeism [13]. Collectively, it is evident that postdromal symptoms are burdensome and can contribute to the overall impact of migraine. This lends support to our findings regarding HIT-6 and WHODAS. The number of postdromal symptoms rather than just their presence might thus be relevant to consider in both clinical and research settings.

### Strengths and limitations

The present study stands as the first to use the ICHD-3 definition for postdromal symptoms in a well-characterized population of adults with migraine [24]. The issue of acquiescence bias was also somewhat accounted for via the use of both prompted and unprompted means of enquiry. However, several limitations should be considered when interpreting our results. First, retrospective assessment carries the risk of recall bias, which can lead to underreporting of postdromal symptoms [9]. Second, the onset, duration, and severity of each postdromal symptoms was not evaluated nor were the side effects experienced when using acute medications. Third, the potential for selection bias must be acknowledged. Our study population was primarily recruited from a tertiary care setting and predominantly diagnosed with chronic migraine. This approach skews our sample towards people with more severe disease manifestations, thereby limiting the extrapolation of our results to the broader migraine population. Lastly, our participants’ high migraine frequency might complicate the clear differentiation of migraine phases, increasing the likelihood of symptom misattribution. However, it is pertinent to note that existing literature suggests that the actual duration of premonitory and postdromal symptoms typically does not reach the 48-h maximum outlined in the ICHD-3 criteria [7, 9, 13–15]. This suggests that the theoretical maximum duration might not accurately reflect the lived reality of most people with migraine, as the occurrence of symptoms persisting for the full 48-h window is relatively rare. Thus, restricting the investigation of premonitory and postdromal symptoms to people with only episodic migraine introduces different biases and limit the generalizability of the findings.

### Perspectives

To more accurately delineate the migraine postdrome and its associated symptoms, it is essential to use standardized case definitions and robust methods of data collection. Prospective, population-based studies, particularly those using electronic diaries with regular entries and time stamps, would be highly beneficial. These studies should adhere to the ICHD-3 definition of postdromal symptoms. In addition, comprehensive recording of each non-headache symptom, including its onset, duration, and severity across all phases of migraine, is important to achieve a more complete understanding of the migraine postdrome. Furthermore, the present findings indicated a positive correlation between the number of postdromal symptoms and the disease burden. This is an area ripe for exploration and might reveal the impact of postdromal symptoms on the disease burden attributed to migraine. To this end, the development of questionnaires specifically tailored to evaluate the burden associated with postdromal symptoms is essential.

## Conclusions

Our study identified a widespread prevalence of postdromal symptoms among adults with migraine from a tertiary care population. These symptoms might be a common disease feature, although confirmation requires population-based data. It is also important to consider that symptom reporting is influenced by the means of enquiry, in addition to the presence of premonitory symptoms and migraine frequency. Future, prospective, observational studies should assess the intricate relationship between differences phases of migraine, as well as the contribution of postdromal symptoms to the overall disease burden.

### Supplementary Information

**Additional file 1.**

## Data Availability

The dataset used and/or analyzed during the current study are available from the corresponding author on reasonable request.

## Abbreviations

BMI: Body Mass Index
CI: Confidence Interval
HIT-6: Six-Item Headache Impact Test
ICHD: International Classification of Headache Disorders
IQR: Interquartile Range
MIDAS: Migraine Disability Assessment
MOH: Medication-Overuse Headache
OR: Odds Ratio
PROMs: Patient-Peported Outcome Measures
REDCap: Research Electronic Data Capture
REFORM: Registry for Migraine
SD: Standard Deviations
WHODAS 2.0: World Health Organization Disability Assessment 2.0
