# Supplementary material for: Postdromal symptoms in migraine: a REFORM study
Source: J Headache Pain. 2024 Feb 21;25(1):25. doi: 10.1186/s10194-024-01716-3 (PMC10880332; doi:10.1186/s10194-024-01716-3)
Supplement: Supplementary file 1 — Additional file 1. [file 10194_2024_1716_MOESM1_ESM.docx]

**Supplementary Appendix**

**Appendix 1. Inclusion and Exclusion Criteria for the Parental Study, REFORM**

*Inclusion criteria:*

- ≥18 years of age at screening
- History of migraine without aura, migraine with aura, or chronic migraine according to the International Classification of Headache Disorders (ICHD) version 3^rd^ edition criteria (code 1.1, 1.2 or 1.3) for ≥1 year prior to study entry
- ≥4 monthly headache days that meet criteria as migraine days on average for three months prior to study screening
- Preventive treatment with erenumab scheduled at the Danish Headache Center, Department of Neurology, University Hospital Rigshospitalet
- Provision of informed consent prior to initiation of any study-related activities/procedures

*Exclusion criteria:*

- >50 years of age at migraine onset
- History of persistent post-traumatic headache, hemiplegic migraine, or cluster headache (ICHD-3 code 5.2, 1.2.3, and 3.1)
- Inability to differentiate migraine headache from other headaches
- Risk of self-harm or harm to others as evidenced by past suicidal behavior
- History or evidence of any other clinically significant disorder, condition, or disease (except those outlined above), that might pose a risk to subject safety or interfere with study evaluation
- Previous treatment with erenumab
- Treatment with another anti-CGRP monoclonal antibody for three months prior to screening
- Concomitant preventive medication apart from anti-CGRP antibodies was allowed, but only if dosage was stable for two months prior to the screening
- Female participants of childbearing potential unwilling to use acceptable methods of contraception (oral contraceptives, intrauterine device, intrauterine hormonal-releasing system, bilateral tubal ligation/occlusion, vasectomized partner, sexual abstinence, male or female condom, cap, diaphragm, or sponge with spermicide)
- Known sensitivity to any products or components to be administered
- Not likely to be able to complete all protocol required study visits or procedures, and/or comply with all required study procedures to the best of the subject and study investigator’s knowledge

**Abbreviations**: CGRP, calcitonin gene-related peptide; ICHD-3: International Classification of Headache Disorders, 3^rd^ edition.
